# Visions of Fancy, Poetical Work

**Published:** 1884-11

**Authors:** 


**Visions of Fancy, Poetical Work.** By N. M. BASKITT, M. D. St. Louis, Mo.: Commercial Printing Co., 405 North Third Street. 1884.

If there is any excuse for the existence of this work, we are unable to find it, after a cursory examination of its contents. There is little poetry in the ordinary routine of a physician's life, and we doubt if this work will relieve the professional reader of any of the cares and anxieties which beset his pathway.

